# *Mycobacterium tuberculosis* H37Rv Strain Increases the Frequency of CD3^+^TCR^+^ Macrophages and Affects Their Phenotype, but Not Their Migration Ability

**DOI:** 10.3390/ijms23010329

**Published:** 2021-12-28

**Authors:** Lucero A. Ramon-Luing, Claudia Carranza, Norma A. Téllez-Navarrete, Karen Medina-Quero, Yolanda Gonzalez, Martha Torres, Leslie Chavez-Galan

**Affiliations:** 1 Laboratory of Integrative Immunology, Instituto Nacional de Enfermedades Respiratorias Ismael Cosío Villegas, Mexico City 14080, Mexico; ramonluing@yahoo.com.mx (L.A.R.-L.); norma.tellez@gmail.com (N.A.T.-N.); 2 Laboratory of Tuberculosis Immunobiology, Instituto Nacional de Enfermedades Respiratorias Ismael Cosio Villegas, Mexico City 14080, Mexico; carranza.salazar.claudia@gmail.com (C.C.); marthatorres98@yahoo.com (M.T.); 3 Laboratory of Immunology, Escuela Militar de Graduados de Sanidad, Mexico City 11200, Mexico; kmq.kmq5@gmail.com; 4 Department of Microbiology, Instituto Nacional de Enfermedades Respiratorias Ismael Cosio Villegas, Mexico City 14080, Mexico; ygonzalezh@iner.gob.mx

**Keywords:** TCR/CD3, TNF-pathway, macrophages, cell migration, *Mycobacterium tuberculosis*

## Abstract

In mycobacterial infections, the number of cells from two newly discovered subpopulations of CD3^+^ myeloid cells are increased at the infection site; one type expresses the T cell receptor (CD3^+^TCRαβ^+^) and the other does not (CD3^+^TCRαβ^−^). The role of *Mycobacterium tuberculosis* (Mtb) virulence in generating these subpopulations and the ability of these cells to migrate remains unclear. In this study, monocyte-derived macrophages (MDMs) infected in vitro with either a virulent (H37Rv) or an avirulent (H37Ra) Mtb strain were phenotypically characterized based on three MDM phenotypes (CD3^−^, CD3^+^TCRαβ^+^, and CD3^+^TCRαβ^−^); then, their migration ability upon Mtb infection was evaluated. We found no differences in the frequency of CD3^+^ MDMs at 24 h of infection with either Mtb strain. However, H37Rv infection increased the frequency of CD3^+^TCRαβ^+^ MDMs at a multiplicity of infection of 1 and altered the expression of CD1b, CD1c, and TNF on the surface of cells from both the CD3^+^ MDM subpopulations; it also modified the expression of CCR2, CXCR1, and CCR7, thus affecting CCL2 and IL-8 levels. Moreover, H37Rv infection decreased the migration ability of the CD3^−^ MDMs, but not CD3^+^ MDMs. These results confirm that the CD3^+^ macrophage subpopulations express chemokine receptors that respond to chemoattractants, facilitating cell migration. Together, these data suggest that CD3^+^ MDMs are a functional subpopulation involved in the immune response against Mtb.

## 1. Introduction

Tuberculosis (TB), an infectious disease caused by *Mycobacterium tuberculosis* (Mtb), remains a serious public health problem worldwide despite the efforts to eradicate it. The World Health Organization reported that in 2021, there were 10 million TB cases and 1.3 million deaths [1]. TB primarily affects the lungs; alveolar macrophages represent the first line of defense for the immune response against Mtb. Macrophages play essential roles in the initial recognition, processing, and presentation of antigens; moreover, they can release proinflammatory cytokines and chemokines to recruit cells to the site of infection [2,3]. Macrophage subpopulations are classified based on the cytokines produced by them in response to stimuli, and M1 macrophages release proinflammatory cytokines, whereas M2 macrophages release cytokines that favor an anti-inflammatory response [4]. In addition to these well-known subpopulations, a new macrophage phenotype bearing the CD3/T cell receptor (TCR) complex has been reported. These macrophages form clusters around the caseous zone of granulomas in patients with TB, and their presence depends on tumor necrosis factor (TNF) [5].

TNF is one of the most important cytokines involved in mycobacterial infections; it is a trimeric molecule expressed on the cell surface (transmembrane TNF [tmTNF]). After an activation stimulus, tmTNF is proteolytically processed via the action of a disintegrin and metalloproteinase domain 17 to release the soluble form of TNF (solTNF). Both tmTNF and solTNF are bioactive molecules that function by interacting with their receptors, 1 or 2 (TNFR1 and TNFR2, respectively), which in turn can also be found in transmembrane or soluble forms [6].

More recently, it has been determined that CD3^+^ macrophages can be classified into two subpopulations, one that expresses CD3 but not TCR (CD3^+^TCRαβ^−^) and another that expresses both CD3 and TCR (CD3^+^TCRαβ^+^). Regarding CD3^+^ macrophages, there are several gaps in the literature; however, at present, we know that cells from both the CD3^+^ macrophage subpopulations release proinflammatory cytokines via a CD3-dependent pathway, and a murine model of mycobacterial pleural infection has demonstrated that the frequency of CD3^+^ myeloid cells increases at the site infection [7]. In this regard, data from other studies have reported that mice infected with BCG have an increased frequency of CD11b^+^CD3^+^TCRαβ^+^ cells, and apparently, TNFR1 plays a critical role in CD11b^+^CD3^+^ cell recruitment [5,8].

Recently, it was reported that heathy donors have approximately 3% of CD3^+^ monocytes in the peripheral blood, whereas patients with pulmonary TB have an increased frequency of this newly discovered subpopulation (13%). More interestingly, the circulating CD3^+^ monocyte frequency had normalized at the sixth month of treatment in patients with TB sensitive to first-line drugs [9]. Thus, a growing body of evidence indicates that the frequency of CD3^+^ myeloid cells increase as a response to mycobacterial infections, and that TNF/TNFR axis plays an essential role in the maintenance of these cells; however, little is known about the specific mechanism used by these cells to migrate to the site of infection. 

Mtb virulence influences diverse macrophage functions to create a favorable environment for the survival of the bacteria. For example, specific proteins of virulent strains increase their sensitivity to an acid medium, reduce cell death, and enhance the production of interleukin (IL)-10 to inhibit the activation of a Th1 response [10,11,12,13]. Other reports have also demonstrated that non-pathogenic strains induce the production of TNF, whereas hypervirulent strains reduce its secretion [10]. Based on all these considerations, it is reasonable to assume that the migration ability of the CD3^+^ macrophages may depend on at least three factors, including the type of chemokines in the microenvironment, the expression of the corresponding receptors on macrophages, and the virulence of the Mtb strain.

A better understanding of this process would be relevant, considering CD3^+^ macrophage subpopulations can participate in the regulation of the local proinflammatory response and therefore, in the control of infection. In this study, we evaluated the role of Mtb virulence in the generation of human CD3^+^TCRαβ^+^ and CD3^+^TCRαβ^−^ monocyte-derived macrophages (MDMs). First, we infected MDMs in vitro for 24 h with two Mtb strains, avirulent H37Ra and virulent H37Rv. Then, we performed a phenotypical characterization of the cells of the subpopulations, specifically CD3^−^ (classical macrophages) cells and CD3^+^TCRαβ^+^ and CD3^+^TCRαβ^−^ MDMs. Finally, we evaluated their ability to migrate in response to the chemokines CCL2, IL-8, and CCL19. 

## 2. Results

### 2.1. Mtb H37Rv Virulent Strain Increases the Expression of TCR on the CD3^+^ MDMs

The frequency of CD3^+^ myeloid cells has been reported to be increased upon mycobacterial infections [7,8,9]. Therefore, to evaluate the effect of Mtb virulence on the induction of these subpopulations, MDMs were infected with H37Ra (multiplicity of infection (MOI) = 1 and 10) and H37Rv (MOI = 1 and 5) for a short time (24 h). The phenotypic characterization by flow cytometry showed that 98% were monocytes (CD14^+^ cells), with minimum contamination of CD2^+^ (1%) and CD19^+^ (2%) cells (Supplementary Figure S1), and after culture for 7 days, MDMs were obtained. Thus, to identify the expression of CD3 and TCRαβ on MDMs, the analysis strategy included a gate of CD2^−^ cells (for the complete exclusion of T cells), and subsequently, the dot plot was divided into two regions according to CD3 expression, specifically CD3^−^ and CD3^+^ gates. Finally, the expression of TCRαβ was determined in the CD3^+^ gate (Supplementary Figure S2). 

**Figure 1:**
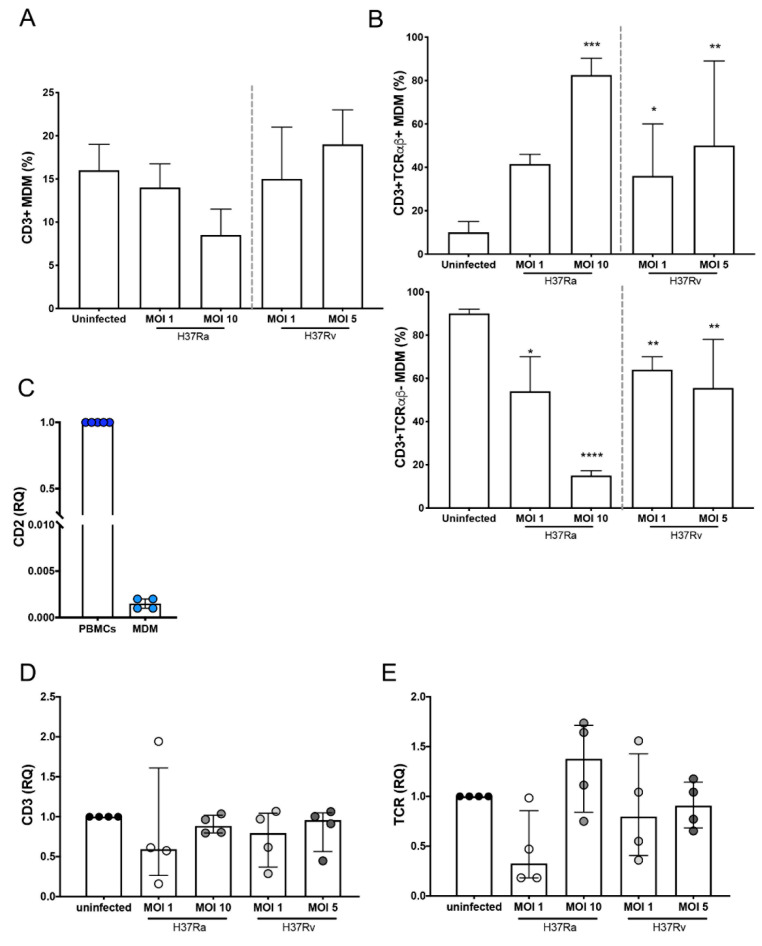
Expression of TCR is increased on the surface of CD3^+^ monocyte-derived macrophages (MDMs) infected with the *Mycobacterium tuberculosis* (Mtb) H37Rv virulent strain. Human MDMs were obtained after 7 days in culture; 2 × 10^6^ MDMs (per condition) were infected, and at 24 h post-infection, they were recovered. (**A**) Frequency of CD3^+^ MDMs among macrophages infected with H37Ra (MOI = 1 and 10) and H37Rv (MOI = 1 and 5) Mtb strains; and (**B**) frequency of CD3^+^TCRαβ^+^ and CD3^+^TCRαβ^−^ subpopulations. The relative expression of *CD2* (**C**), *CD3* (**D**), and *TCR* (**E**) genes in Mtb-infected MDMs was assessed by real-time qPCR, and the gene expression was calculated relative to that in the uninfected MDMs for each experimental replicate (RQ = 1). Bar graphs in A and B show median values and interquartile ranges (IQRs, 10–90) from four independent experiments; scatter plots with bar in C, D, and E show median values and IQRs from four independent experiments. Statistical analysis was performed with the Kruskal–Wallis test followed by Dunn’s post-test to compare infected versus uninfected MDMs. * *p* < 0.05, ** *p* < 0.01, *** *p* < 0.001, **** *p* < 0.0001.

The frequency of CD3^+^ MDMs was ~15% in the uninfected condition, and after 24 h of infection, H37Ra and H37Rv, at high or low MOIs, did not affect this frequency (Figure 1A). However, 24 h of infection was enough to increase the frequency of CD3^+^ MDMs expressing TCRαβ; only a MOI of 10 of the avirulent H37Ra strain increased the frequency of CD3^+^TCRαβ^+^ MDMs, whereas for the virulent H37Rv strain, the increase was observed at both MOIs of 1 and 10 (Figure 1B, upper). The increase in the frequency of CD3^+^TCRαβ^+^ MDMs was associated with a corresponding decrease in the frequency of CD3^+^TCRαβ^−^ MDMs (Figure 1B, lower). 

To further confirm that the MDMs obtained in our system were not lymphoid cells, the expression of CD2 was evaluated at the transcript level. There was almost no expression of *CD2* in MDMs, unlike its expression in peripheral blood mononuclear cells (PBMCs; Figure 1C). As observed by flow cytometry, the expression of CD3 was not modified at the transcript level during infection with both H37Ra and H37Rv (Figure 1D), whereas the expression of the epsilon chain of the TCR showed a discrete increase with infection compared with that in uninfected cells (Figure 1E). Together, our data suggest that the Mtb virulent strain does not increase the frequency of CD3^+^ MDMs, at least during the first 24 h of infection; however, TCRαβ expression on the MDMs is induced more efficiently by the virulent strain.

### 2.2. Antigen-Presenting Molecules Are Differentially Modified on the Surface of CD3^+^TCRαβ^−^ and CD3^+^TCRαβ^+^ MDMs

Human leukocyte antigen (HLA) molecules are necessary to present antigens of protein origin to T cells. Specifically, HLA class II (HLA-II) interacts with CD4^+^ T cells, whereas the CD1 family presents antigens of non-protein origin to diverse T cell subpopulations. The isoforms CD1b and CD1c have been suggested as molecules involved in the Mtb antigen presentation process [14,15]. Thus, we evaluated the effect of Mtb infection on the expression of MHC-II, CD1b, and CD1c in CD3^−^, CD3^+^TCRαβ^+^, and CD3^+^TCRαβ^−^ MDMs by flow cytometry (Supplementary Figure S2).

**Figure 2:**
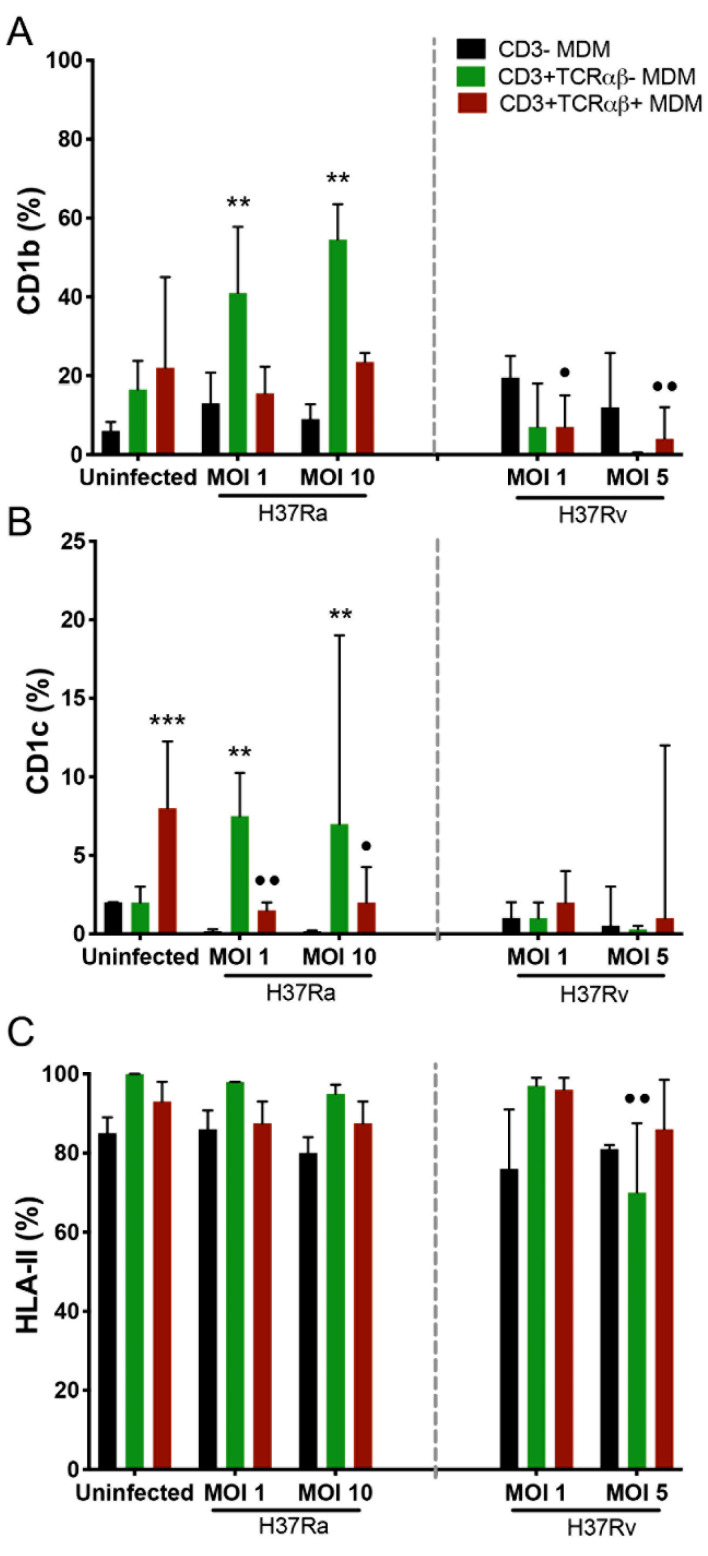
Virulence of the *Mycobacterium tuberculosis* (Mtb) strain modulates the expression of antigen-presenting molecules on CD3^+^TCRαβ^+^ and CD3^+^TCRαβ^−^ monocyte-derived macrophage (MDM) subpopulations. After 7 days in culture, 2 × 10^6^ MDMs (per condition) were infected, and at 24 h post-infection, they were recovered for flow cytometric analysis. Frequency of Mtb-infected MDM subpopulations expressing CD1b (**A**), CD1c (**B**), and HLA-II (**C**) molecules. Bar graphs show median values and interquartile ranges (IQRs, 10–90) from four independent experiments. Kruskal–Wallis tests followed by Dunn’s post-tests were performed to compare macrophage subpopulations under the same MOI conditions versus CD3^−^ MDM subpopulation, ** *p* < 0.01, *** *p* < 0.001, and to compare the infected macrophage subpopulation versus its counterpart under uninfected conditions, • *p* < 0.05, •• *p* < 0.01.

The Mtb virulent strain differentially modified the expression of the antigen-presenting molecules. The frequency of CD3^+^TCRαβ^−^CD1b^+^ and CD3^+^TCRαβ^−^CD1c^+^ MDMs was increased upon infection with the H37Ra strain (at both MOIs) compared with the frequency of CD3^−^ MDMs (asterisks). In contrast, only the CD3^+^TCRαβ^+^CD1c^+^ MDM frequency was affected, as compared with the frequency of the same subpopulation under the uninfected condition (dots; Figure 2A,B, left). However, infection with the H37Ra strain did not affect HLA-II expression on the MDM subpopulations (Figure 2C, left).

The virulent H37Rv strain (at both MOIs) decreased the frequency of CD3^+^TCRαβ^+^CD1b^+^ MDMs compared with that in the uninfected condition (dots), without affecting the expression of the CD1c isoform (Figure 2A and B, right, respectively). In contrast, infection with H37Rv decreased the frequency of the CD3^+^TCRαβ^−^HLA-II^+^ MDM subpopulation compared with the frequency of the same subpopulation in the uninfected condition (Figure 2C, right). The results showed that the avirulent Mtb strain increased the expression of antigen-presenting molecules on CD3^+^TCRαβ^−^ MDMs; in contrast, the virulent Mtb strain decreased the expression of CD1b on CD3^+^TCRαβ^+^ and CD3^+^TCRαβ^−^ MDMs.

### 2.3. H37Rv Virulent Mtb Strain Decreases the Expression of tmTNF, and H37Ra Avirulent Mtb Strain Increases the Expression of tmTNF and tmTNFR1 on CD3^+^TCRαβ^−^ and CD3^+^TCRαβ^+^ MDMs

**Figure 3:**
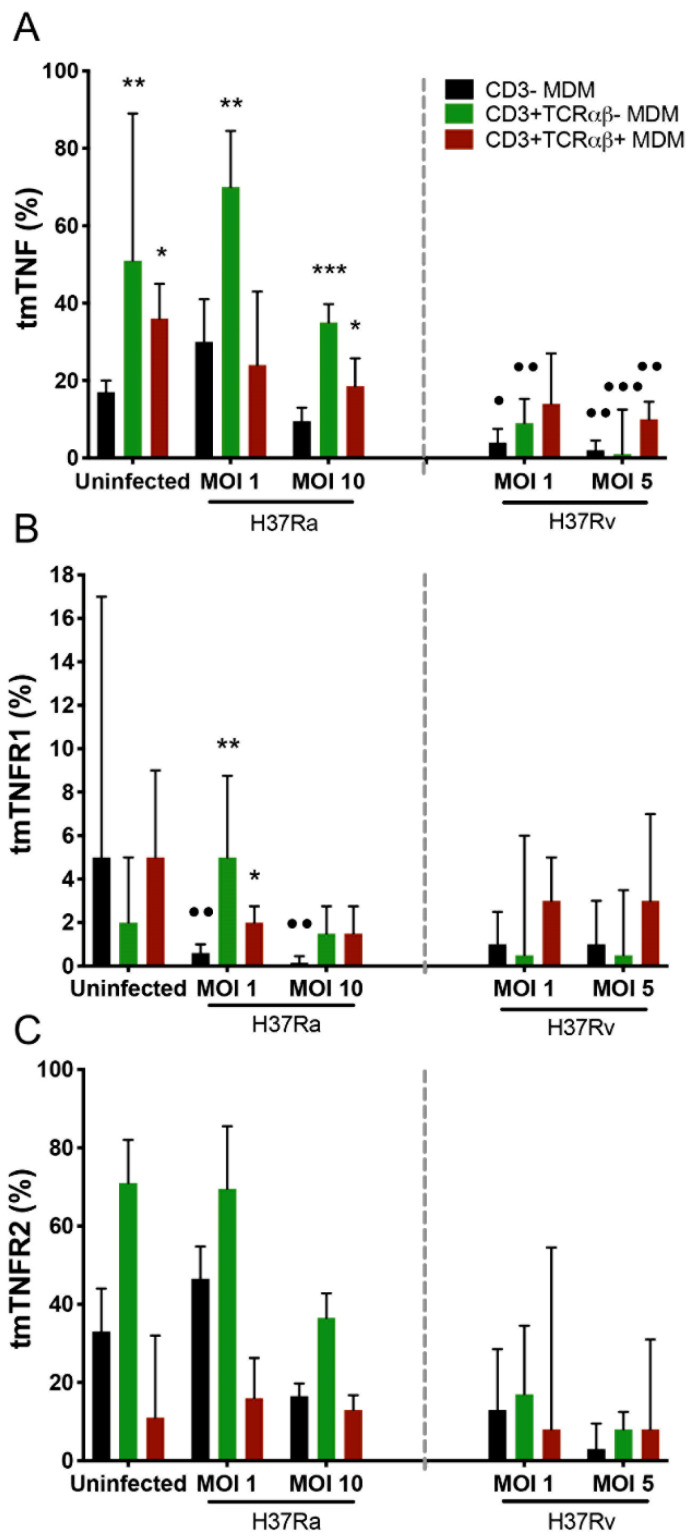
Expression of tmTNF on myocyte-derived macrophages (MDMs) infected with the *Mycobacterium tuberculosis* (Mtb) H37Rv virulent strain decreased, whereas that of only tmTNFR1 significantly decreased in MDMs infected with the Mtb H37Ra avirulent strain. After 7 days in culture, 2 × 10^6^ MDMs (per condition) were administered, and at 24 h post-infection, they were recovered for flow cytometric analysis. Frequency of Mtb-infected MDM subpopulations expressing tmTNF (**A**), tmTNFR1 (**B**), and tmTNFR2 (**C**) molecules. Bar graphs show median values and interquartile ranges (IQRs, 10–90) from four independent experiments. Kruskal–Wallis tests followed by Dunn’s post-tests were performed to compare macrophage subpopulations under the same MOI conditions versus the CD3^−^ MDM subpopulation, * *p* < 0.05, ** *p* < 0.01, *** *p* < 0.001, and, to compare the infected macrophage subpopulation versus its counterpart under uninfected conditions, • *p* < 0.05, •• *p* < 0.01, ••• *p* < 0.001.

The participation of the TNF pathway in CD3^+^ myeloid cells has been reported in different models of mycobacterial infection [5,7,8]. The expression of tmTNF, tmTNFR1, and tmTNFR2 inside the gate of CD3^−^, CD3^+^TCRαβ^+^, and CD3^+^TCRαβ^−^ MDMs was evaluated by flow cytometry (Supplementary Figure S2). Compared with the frequency of CD3^−^ MDMs, H37Ra increased (asterisks) the frequency of CD3^+^TCRαβ^−^tmTNF^+^ MDMs at an MOI of 1 and of both CD3^+^TCRαβ^−^tmTNF^+^ and CD3^+^TCRαβ^+^tmTNF^+^ MDMs at an MOI of 10; here, it is important to note that, in the uninfected condition, the frequency of CD3^+^TCRαβ^−^tmTNF^+^ and CD3^+^TCRαβ^+^tmTNF^+^ MDMs was higher than that of CD3^−^ MDMs (Figure 3A, left). Regarding the expression of TNFRs, H37Ra at an MOI of 1 increased the frequency of CD3^+^TCRαβ^−^tmTNFR1^+^ and CD3^+^TCRαβ^+^tmTNFR1^+^ MDMs compared with that of CD3^-^tmTNFR1^+^ MDMs (asterisks). Interestingly, when the CD3^−^tmTNFR1^+^ MDM frequency generated during the H37Ra infection was compared with that in the uninfected condition, the subpopulation frequency was decreased (dots; Figure 3B, left), and the expression of tmTNFR2 was not affected by infection with H37Ra and H37Rv (Figure 3C). 

The virulent H37Rv strain at an MOI of 1 decreased the frequency of only CD3^−^tmTNF^+^ and CD3^+^TCRαβ^−^tmTNF^+^ MDMs. Meanwhile, at an MOI of 5, it decreased both CD3^+^TCRαβ^−^tmTNF^+^ and CD3^+^TCRαβ^+^tmTNF^+^ MDM subpopulations compared with their respective frequencies under the uninfected condition (dots; Figure 3A, right). We did not identify any alteration in the expression of tmTNFR1 and tmTNFR2 on the MDM subpopulations upon H37Rv infection (Figure 3B and C, right, respectively). 

### 2.4. Infection with the Virulent Mtb Strain Increases the Level of solTNF and solTNFR2, but Not solTNFR1, in MDM Subpopulations That Favor a Proinflammatory Microenvironmen

**Figure 4:**
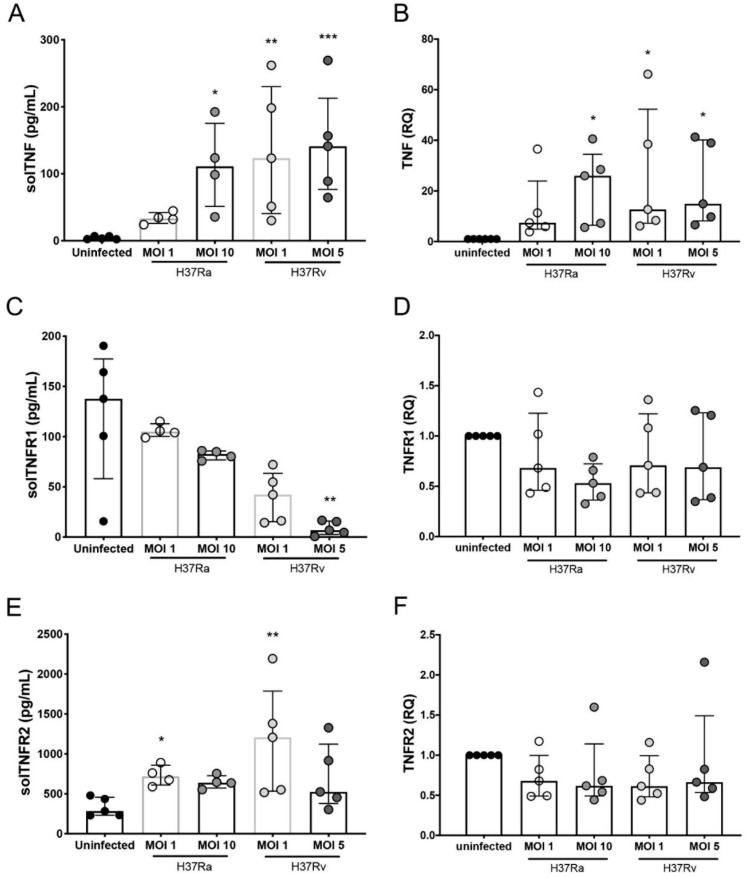
Infection with *Mycobacterium tuberculosis* (Mtb) H37Rv induces differential expression at the transcript level and the secretion of TNF/TNFR axis molecules in monocyte-derived macrophages (MDMs). Supernatants of MDMs infected with H37Ra (MOI = 1 and 10) and H37Rv (MOI = 1 and 5) Mtb strains at 24 h post-culture (at a ratio of 1 × 10^6^ MDMs/mL) were recovered for ELISA, and MDMs were used for qPCR assays. Levels of soluble (sol) TNF (**A**), TNFR1 (**C**), and TNFR2 (**E**), as well the relative expression of *TNF* (**B**), *TNFR1* (**D**), and *TNFR2* (**F**) genes, were evaluated in supernatants and Mtb-infected MDMs (respectively). The gene expression was calculated relative to that in the uninfected MDMs for each experimental replicate (RQ = 1). Scatter plots with bar show median values and interquartile ranges (IQRs, 10–90) from five independent experiments. Statistical analysis of soluble levels and gene expression was performed using the Kruskal–Wallis test followed by Dunn’s post-test to compare infected versus uninfected MDMs, * *p* < 0.05, ** *p* < 0.01, *** *p* < 0.001.

The expression of tmTNF and tmTNFR1 on the CD3^+^ MDM subpopulations was differentially regulated in a virulence-dependent manner and mediated a proinflammatory microenvironment, which helps activate diverse functions that protect against Mtb infection through interactions with its receptors [6]. We evaluated the levels of solTNF, solTNFR1, and solTNFR2 produced by MDMs infected with H37Ra or H37Rv. H37Ra increased only the solTNF levels at the higher MOI, whereas the level of solTNFR2 was increased at a MOI of 1, and that of solTNFR1 was unaffected (Figure 4A, E and C, left, respectively). In contrast, H37Rv infection increased the solTNF levels at both MOIs and solTNFR2 levels at the lower MOI; interestingly, the levels of solTNFR1 were decreased only at MOI 5 (Figure 4A, E and C, right, respectively). With H37Rv infection, tmTNF expression on the surface of all MDM subpopulations was decreased, but that of solTNF was increased (Figure 3A and Figure 4A, right, respectively). 

We hypothesized that MDMs produce high levels of TNF; however, during the virulent Mtb strain infection, it sheds from the cell surface, and consequently, the tmTNF levels are decreased, whereas those of solTNF are increased. To confirm our hypothesis, we evaluated the expression of *TNF*, *TNFR1*, and *TNFR2* at the transcript level in MDM infected with H37Ra and H37Rv. Our data confirmed that H37Ra infection increased the expression of TNF at the transcription level, but only at the higher MOI, whereas H37Rv did so at an MOI of 1 (Figure 4B). Furthermore, despite the changes observed in the transmembrane and soluble forms of TNFRs, we did not observe any change in the transcript levels of *TNFR1* or *TNFR2* (Figure 4D and F, respectively). 

The proinflammatory response during Mtb infection is mediated by a variety of cytokines rather than through TNF [16,17]. Therefore, to evaluate a more integral microenvironment, the levels of proinflammatory proteins, namely interferon-gamma (IFN-γ) interferon-gamma inducible protein-10 (IP-10), and the classical anti-inflammatory interleukin (IL)-10, were measured. H37Ra only induced the production of IL-10 at the low MOI; however, H37Rv was able to induce the production of IL-10, IFN-γ, and IP-10 (Supplementary Figure S3). In summary, the proinflammatory environment created by MDMs is favored mainly during infection with a virulent Mtb strain.

### 2.5. Virulence of Mtb Affects the Expression of Chemokine Receptors on CD3^−^ and CD3^+^ MDM Subpopulations

The activation of the chemokine/chemokine receptor axis is one of the most important processes in the regulation of cell migration [18]. Recently, we reported that CD3^+^ monocytes from patients with TB poorly express CCR2; nevertheless, they are able to migrate in response to IP-10 stimuli [9]. Therefore, the expression of CCR2, CXCR1, and CCR7 chemokine receptors inside the gate of CD3^−^, CD3^+^TCRαβ^+^, and CD3^+^TCRαβ^−^ MDMs was evaluated by flow cytometry, and that of CCL2 (ligand to CCR2), IL-8 (ligand to CXCR1), and CCL19 (ligand to CCR7) was evaluated in the culture supernatant by ELISA. 

**Figure 5:**
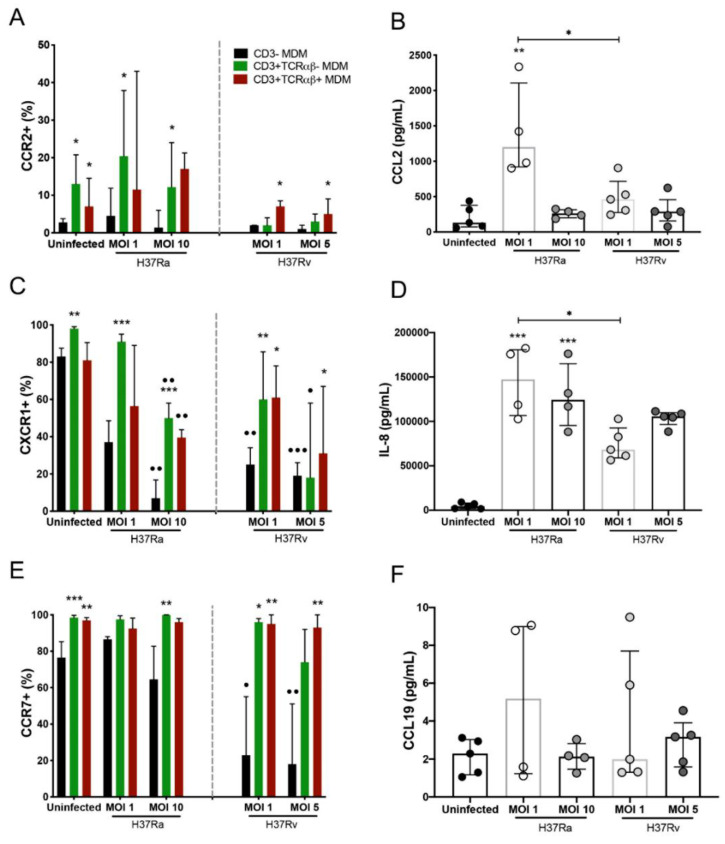
Chemokine receptors and their ligands implicated in the host inflammatory response are differentially expressed in *Mycobacterium tuberculosis* (Mtb) H37Ra- or H37Rv-infected CD3^−^ and CD3^+^ monocyte-derived macrophages (MDMs). After 7 days in culture, 2 × 10^6^ MDMs (per condition) were infected, and at 24 h post-infection, MDMs were recovered for flow cytometric analysis, and the supernatants were recovered for ELISA. (**A**) CCR2, (**C**) CXCR1, and (**E**) CCR7 chemokine receptors were quantified by flow cytometry. Soluble levels of the (**B**) CCL2, (**D**) IL-8, and (**F**) CCL19 chemokines were assessed in culture supernatants by ELISA. Bar graphs in A, C, and E show median values and interquartile ranges (IQRs, 10–90) from four independent experiments; scatter plots with bar in B, D, and F show median values and IQRs from five independent experiments. Kruskal–Wallis tests followed by Dunn’s post-tests with bar were performed to compare macrophage subpopulations under the same MOI conditions versus the CD3^−^ MDM subpopulation, * *p* < 0.05, ** *p* < 0.01, *** *p* < 0.001, and to compare the same macrophage subpopulation in a different condition of culture, • *p* < 0.05, •• *p* < 0.01, ••• *p* < 0.001. In addition, for the analysis of soluble chemokine levels, the Kruskal–Wallis test followed by Dunn’s post-test was performed to compare infected versus uninfected MDMs or between groups (when indicated), * *p* < 0.05, ** *p* < 0.01, *** *p* < 0.001.

Compared to that of CD3^−^ MDMs, H37Ra (MOI 1) increased the frequency of CD3^+^TCRαβ^−^CCR2^+^ MDMs, as well as their ligand, CCL2 (Figure 5A,B, left, respectively), whereas H37Rv infection (at both MOIs) induced an increase in the frequency of CD3^+^TCRαβ^+^CCR2^+^ MDMs compared with that of CD3^−^ MDMs (asterisk), and the CCL2 levels were similar (Figure 5A,B, right, respectively), although they were lower than those with H37Ra infection. Compared with that of CD3^−^ MDMs, the frequency of CD3^+^TCRαβ^−^CXCR1^+^ and CD3^+^TCRαβ^+^CXCR1^+^ MDMs was increased in both strains. However, the frequency of CD3^−^ MDMs decreased compared to that in the uninfected condition (dots; Figure 5C). Regarding levels of IL-8, the ligand of CXCR1, we observed that H37Ra and H37Rv induced the production of IL-8, but the H37Ra strain induced higher production than H37Rv (Figure 5D). Furthermore, H37Rv affected the frequency of CD3^−^CCR7^+^ MDMs compared with that in the uninfected condition (dots). In contrast, the frequencies of CD3^+^TCRαβ^−^CCR7^+^ and CD3^+^TCRαβ^+^CCR7^+^ MDMs were higher than that of CD3^−^CCR7^+^ MDMs (asterisks; Figure 5E), and CCL19 levels (ligand to CCR7) were not modified as a result of H37Ra and H37Rv infection (Figure 5F). Together, these data show that the virulence of Mtb affects the expression of chemokine receptors, as well as CCL2 and IL-8, negatively, suggesting that CD3^+^ MDM subpopulations have altered migration ability.

### 2.6. Virulence of Mtb affects the Migration Capacity of MDMs, but the CD3^+^ MDM Subpopulation Maintains Its Migration Ability

**Figure 6:**
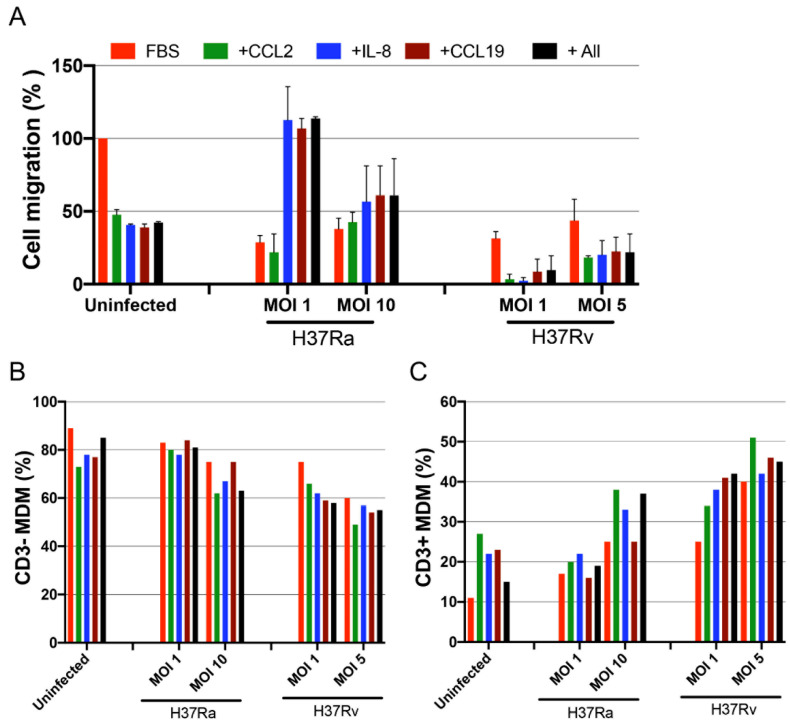
The migration ability of CD3^+^ monocyte-derived macrophages (MDMs) is modulated depending on *Mycobacterium tuberculosis* (Mtb) virulence. MDMs infected with H37Ra (MOI = 1 and 10) or H37Rv (MOI = 1 and 5) Mtb strains were recovered 24 h post-infection to evaluate their migration ability in response to CCL2, IL-8, and CCL19 chemoattractants using a quantitative fluorometric assay. A 100 µL suspension of infected MDMs (4 × 10^5^) was added to each well. (**A**) Cell migration percentage of Mtb-infected MDMs from three donors was calculated; uninfected MDMs in the presence of the attractant 10% fetal bovine serum were used as a reference (100%). The frequencies of transmigrated cells analyzed by flow cytometry (**B**), percentage of transmigrated CD3^−^ MDMs, and (**C**) CD3^+^ MDM subpopulations are shown. Bar graphs show median values and interquartile ranges (IQRs, 10–90) from three independent experiments.

To assess the migration ability of the novel CD3^+^ MDM subpopulation, we evaluated the migration of MDM cells, infected with H37Ra and H37Rv (24 h), in response to the chemokines CCL2, IL-8, and CCL19. MDMs infected with H37Ra (MOI = 1) exhibited a high ability to migrate in response to IL-8, CCL19, and a combination of the three chemokines. However, these MDMs lost their capacity to respond to CCL2. H37Ra (MOI = 10) decreased the migration ability by approximately 50% compared with that in uninfected MDMs, and these cells did not respond to either the chemokine or positive control stimuli (Figure 6A, middle). In contrast, the H37Rv strain negatively affected the migration ability of MDMs even at an MOI of 1, with these cells losing approximately 80% of their migration ability in response to the chemokine and positive control stimuli (Figure 6A, right). 

Interestingly, evaluation of the expression of CD3 on MDMs that migrated in response to chemokines (Supplementary Figure S4) by flow cytometry revealed that the migration ability of CD3^−^ MDMs was decreased after H37Rv infection, whereas that of CD3^+^ MDMs was retained even after infection with H37Ra and H37Rv (Figure 6B,C). Furthermore, the CD3^+^ MDM prevalence strongly increased after infection with the virulent H37Rv strain compared with that in the uninfected population; the migration of CD3^+^ MDMs increased by more than 100% in the presence of any stimuli (Figure 6C). In conclusion, although Mtb virulence affected the ability of MDMs to migrate, CD3^+^ MDMs not only retained this ability but also showed increased migration when exposed to a virulent strain.

## 3. Discussion

Macrophage subpopulations expressing CD3, with or without TCRαβ, have been reported in both humans and mice. Apparently, these subpopulations perform specific functions, mainly to induce an inflammatory environment [5,7,19,20]. Therefore, to understand the varied roles of macrophages during mycobacterial infection, it is important to investigate the developmental origin and specific phenotypes of macrophages [21]. Here, we detail how the frequency and phenotype of CD3^+^TCRαβ^−^ and CD3^+^TCRαβ^+^ MDM subpopulations are modified after infection with avirulent (H37Ra) and virulent (H37Rv) Mtb strains and how this could determine their migration ability.

We observed that infection with the H37Rv or H37Ra strains did not affect the expression of CD3 on MDMs. However, infection with the virulent H37Rv strain or high doses of the avirulent H37Ra strain increased the percentage of CD3^+^ MDMs that expressed TCRαβ. The role of the CD3^+^TCRαβ^+^ MDM subpopulation in the proinflammatory state and that of TCRαβ^+^ macrophages in the modulation of phagocytosis and macrophage responses during the initial phase of mycobacterial infection has previously been demonstrated [5,7,19]. However, despite the importance of CD3^+^TCRαβ^+^ MDMs in mycobacterial pathogenesis, a complete characterization of this cell population has not been undertaken.

We also evaluated the expression of antigen-presenting molecules, MHC-II, CD1b, and CD1c, on CD3^+^TCRαβ^+^ and CD3^+^TCRαβ^−^ MDM subpopulations and found that the virulent H37Rv strain affects the expression of members of the CD1 family. These results are relevant in the context of TB infection because Mtb has a cell wall rich in lipids, and the CD1 family has been associated with the anti-mycobacterial response [21]. For example, there is evidence indicating that subjects exposed to Mtb antigens have a high frequency of CD1b-specific T cells, which apparently is necessary to properly activate an adaptive immune response against Mtb [22,23]. In this regard, it has been reported that the level of a CD1c^+^ dendritic cell (DC) subset, with a phenotype of CD1c CD19^−^ CD11b^+^, is significantly increased in the peripheral blood from patients with TB. Furthermore, this subpopulation promotes the differentiation of Th17 cells [24]. In contrast, CD1b localizes to the most acidic endocytic compartments, whereas CD1c accumulates on the cell surface and in early endosomes [25]. Thus, the modulation of the expression of CD1 on the CD3^+^ MDM subpopulation might be associated with the evasion mechanisms of the virulent H37Rv strain. 

Because it has been demonstrated that patients receiving TNF antagonists are at a higher risk of developing tuberculosis [25], we evaluated the expression of members of the TNF pathway on CD3^+^ MDMs infected with virulent and avirulent Mtb strains and observed differences associated with virulence. The avirulent H37Ra strain induced CD3^+^TCRαβ^−^ and CD3^+^TCRαβ^+^ MDMs expressing tmTNF and TNFR1. In contrast, the virulent H37Rv strain decreased the CD3^+^TCRαβ^−^tmTNF^+^ and CD3^+^TCRαβ^+^tmTNF^+^ MDM subpopulations. In this regard, both solTNF and tmTNF are required to maintain the immune pressure to contain Mtb reactivation even after establishing mycobacteria-specific immunity. sTNFR2, through the neutralization of TNF, downregulates the expression of IL-12p40 after Mtb infection of DCs [26]. IL-12p40 is required to initiate the control of Mtb, and its deficiency predisposes the host to tuberculosis infection [27]. TNF and TNFR are well-known mediators of the critical actions required for controlling Mtb infection, and the increase in solTNF and sTNFR2 levels could be associated with an evasion pathway induced by the virulent H37Rv strain. 

Previously, we reported that the levels of circulating CD3^+^ monocytes are increased in patients with multidrug-resistant or drug-sensitive TB compared with levels in healthy subjects. Interestingly, although these CD3^+^ monocytes display the ability to migrate, they do not strongly express CCR2 [9]. It is known that granulomas in patients with TB are rich in CD3^+^ macrophages. In a BCG-infection model of mice, we showed the presence of CD3^+^ myeloid cells at the infection site [5,7,8]. Thus, these data prompt the new question of whether these monocytes are recruited in the tissue through a CCR2-independent mechanism. Identifying a unique profile of chemokine receptors in CD3^+^TCRαβ^−^ and CD3^+^TCRαβ^+^ MDMs could be helpful in exploring some hypotheses that could explain their migration pattern into damaged/infected tissue.

As the migration of cells to the lung is an important feature of the innate immune response for the control of microorganisms, our group previously reported that CD3^+^ monocytes from patients with TB are weak regulators of CCR2 in vitro but can migrate in response to MCP-1 and CCL-17 [9]. Therefore, in the present study, we evaluated the effect of Mtb virulence on the expression and migration patterns in CD3^+^ MDMs. We found that the frequency of CD3^+^ MDM subpopulations that expressed CXCR1 was increased after exposure to either H37Rv and H37Ra. Although the frequency of the most abundant subpopulation, CD3^−^ MDMs, decreased, our results are consistent with a previous report indicating the reduced expression of CXCR1 on the granulocytes of patients with pulmonary TB [28]. In addition, H37Rv infection decreased IL-8 levels compared to levels induced by H37Ra. This decrease in the production of IL-8 and CCL-2 suggests compromised migration ability. However, the migration of CD3^+^ MDMs observed in response to IL-8 and CCL19 demonstrates that this ability was not affected by the virulent H37Rv strain. Thus, we speculate that the migration of CD3^+^ MDMs is indeed dependent on integrins, which are essential cell-adhesion receptors involved in the migration of monocytes and macrophages [29].

## 4. Materials and Methods

### 4.1. Ethics Statement

PBMCs were isolated from the buffy coats of 15 donors attending the blood bank at the Instituto Nacional de Enfermedades Respiratorias Ismael Cosío Villegas in Mexico City. Ethical approval for the study was obtained from the Institutional Review Board (IRB# B04-20, approved on 1 February 2020), and the study was conducted according to the principles of the Declaration of Helsinki. Written informed consent for participation was not required for this study in accordance with the national legislation and institutional requirements.

### 4.2. Enrichment of CD14^+^ Cells and Generation of MDMs

PBMCs were isolated from buffy coats using the standard LymphoprepTM (Accurate Chemical-Scientific, Westbury, NY, USA) gradient centrifugation. For the enrichment of monocytes, PBMCs were cultured for 2 h in a Corning^®^ 100 mm TC-treated culture dish (Corning, New York, NY, USA) and allowed to adhere. Non-adherent cells were removed by washing, and adhered cells were recovered using a cell scraper, and positive selection was performed using magnetic microbeads coated with an anti-CD14 monoclonal antibody (Miltenyi Biotech, Bergisch Gladbach, Germany). The purity of the CD14^+^ cell fraction was analyzed by flow cytometry using anti-human CD14, CD2, and CD19 monoclonal antibodies (mAbs) procured from BioLegend (San Diego, CA, USA). The enrichment efficiency of the CD14^+^ cell fraction was routinely >96%, as analyzed by flow cytometry (Figure S1). To obtain MDMs, CD14+ cells were cultured in Costar^®^ 6-well plates at a density of 2 × 10^6^ cells/well (Corning, New York, NY, USA) in RPMI-1640 culture medium (GIBCO, Grand Island, NY, USA), supplemented with 2 mM L-glutamine (GIBCO), 100 µg/mL streptomycin, 100 IU/mL penicillin, and 10% fetal bovine serum (FBS, GIBCO) for 7 days at 37 °C, in an atmosphere of 5% CO_2_. After that, MDMs were recovered and characterized by immunophenotyping based on the expression of differentiation molecules, as previously reported [7,30].

### 4.3. In Vitro MDM-Mtb Infection Assays

As previously reported [31], Mtb laboratory strain stocks (H37Rv, ATCC25618; H37Ra ATCC25177, Manassas, VA, USA) were prepared in Middlebrook 7H9 broth medium (Difco Laboratories, Detroit, MI, USA), supplemented with 10% albumin, dextrose, catalase (BD Biosciences, San Jose, CA, USA), and 0.2% glycerol. After 21 days of incubation at 37 °C, the mycobacterial stock solution was harvested, aliquoted, and stored at −70 °C until use for in vitro infection studies. Colony-forming units (CFUs) in the Middlebrook 7H9 broth medium (Sigma-Aldrich, Fluka, MO, USA) were enumerated after the disruption of mycobacterial clumps. 

A suspension of bacteria was prepared for the infection of macrophages by thawing an aliquot of bacteria and centrifuging it at 6000× *g* for 5 min. Next, the obtained bacterial pellet was resuspended in RPMI medium containing 10% human serum; after that, mycobacteria were declumped by shaking the suspension with three glass beads (3 mm) for 5 min and centrifuged at 800× *g* for 2 min. The single-cell suspensions of bacteria were used for MDM infection. MDMs (2 × 10^6^/mL) were infected with H37Ra at MOIs of 1 and 10 (1 cell per 1 or 10 bacilli, respectively) and with H37Rv at MOIs 1 and 5 (1 cell per 1 or 5 bacilli, respectively). The infected MDMs were incubated at 37 °C for 2 h, and non-phagocytosed bacteria were eliminated by washing; MDMs were further incubated for 24 h at 37 °C.

### 4.4. Assay of Cell Surface Markers by Flow Cytometry

To evaluate the expression of cell surface markers, infected MDMs were recovered and stained for 30 min at 4 °C with monoclonal antibodies against CD14, HLA-DR II, CD1b, CD1c, TCRαβ chain, CD3ε (epsilon chain), CCR4, CCR2, CXCR1, TNFR1, TNFR2, TNF, and CD2 (BioLegend, San Diego, CA, USA). After staining, the cells were washed and fixed with 2% formaldehyde in phosphate-buffered saline (pH 7.2). Data were acquired using a FACS Aria II (BD Biosciences, San Jose, CA, USA) and analyzed using FlowJo v10.2 (Tree Star, San Carlos, CA, USA). For each condition, 50,000 events were generally acquired per sample. Details of the antibodies are included in Supplementary Table S1.

### 4.5. Analysis of Gene Expression by Quantitative Real-Time PCR

Infected MDMs (2 × 10^6^) were recovered after in vitro infection assays, suspended in DNA/RNA Shield solution (Zymo Research, Irvine, CA, USA), and stored at −70 °C until used for RNA extraction. Total RNA was extracted using the RNeasy Micro Kit (Qiagen, Hilden, Germany) according to the manufacturer’s instructions. Genomic DNA contamination was eliminated using an RNA-Free DNAse Set (Qiagen). RNA concentration was determined using the Qubit™ assay kit and the Qubit 2.0 Fluorometer (Life Technologies, Waltham, MA, USA). Total RNA (200 ng) was converted to cDNA using the High-Capacity cDNA Reverse Transcription Kit (Applied Biosystems, Waltham, MA, USA) in a reaction volume of 20 µL following the manufacturer’s guidelines. Gene expression was assessed by quantitative real-time PCR (qPCR) using TaqMan probes for the target genes, including *TNF* (Hs00174128_m1), *TNFR1* (Hs01042313_m1), *TNFR2* (Hs00961750_m1), *CD3* (*CD3e* gene; Hs01062241_m1), *CD2* (Hs01040179_g1), and *TCRab* (*TRBC1* gene; Hs01588269_g1); 18S (18S ribosomal RNA gene) (Hs03928990_g1) and *ACTB* (β-actin) (Hs01060665_g1) were used as endogenous controls. Single reactions were prepared using the Maxima Probe/ROX qPCR Master Mix (Thermo Fisher Scientific, Waltham, MA, USA). Amplifications were performed in duplicate under the following thermal cycling conditions: 95 °C for 10 min, followed by 40 cycles of 60 °C for 1 min and 95 °C for 15 s in the Step One Plus Real-Time PCR System (Applied Biosystems, Waltham, MA, USA). The relative quantification (RQ) of gene expression was determined using the ΔΔCT method to calculate the n-fold change for each target gene under each experimental condition. Results were normalized to levels of endogenous controls, *ACTB* and 18S, and relative to the reference group, uninfected-MDM (where RQ = 2^−ΔΔCT^ =1).

### 4.6. Quantitation of Molecules by ELISA

Culture supernatants of 1 × 10^6^ MDMs/mL (per condition) were collected after 24 h of infection and stored at −70 °C until the analysis. The concentrations of TNF, TNFR1, TNFR2, IL-10, IP-10, CCL2, IL-8, and CCL19 were measured using a sandwich-type immunoassay (ELISA) according to the manufacturer’s instructions. Briefly, ELISA microplates with 96 wells were coated overnight at 4 °C with one of the capture antibodies specific to TNF, IL-10, IFN-γ, CCL2, IL-8 (BioLegend, San Diego, CA, USA), TNFR1, or TNFR2 (R&D Systems, Minneapolis, MN, USA); for CCL19, a kit with pre-coated 96-well microplates was used (Thermo Fisher Scientific, Waltham, MA, USA). Plates were blocked with 1% FBS in PBS for 1 h at room temperature (RT), after which the supernatants were added and incubated for 2 h at RT. Each protein was recognized using a specific biotinylated antibody. Quantification was performed using avidin–HRP conjugate for each ELISA kit, and the tetramethylbenzidine colorimetric substrate was used to develop the blue color. The optical density (450 nm) was measured using a microplate reader (Imark, Bio-Rad, Hercules, CA, USA).

### 4.7. Transwell Cell Migration Assay

The migration of macrophages was assessed based on previously standardized assays [9] and manufacturer recommendations regarding the working concentration range for each chemokine condition reported in the literature [32,33,34]. The chemoattractants, CCL2 (100 ng/mL), CCL19 (100 ng/mL), and IL-8 (150 ng/mL; Peprotech, Cranbury, NJ, USA), were diluted in 150 μL of RPMI medium. A single chemokine or a mixture of the three chemokines was added to the bottom well in a 96-well chemotaxis plate (Cytoselect 96-well cell migration assay-fluorometric format, Cell Biolabs, Inc., San Diego, CA, USA). MDMs were recovered at 24 h post-infection and suspended in RPMI-1640 serum-free medium at a concentration of 4 × 10^5^ cells/100 μL, and this suspension was added to each well on top of the membrane (5 μm pore size) chamber (in the middle of the migration plate). Cells were incubated for 3 h at 37 °C in an atmosphere of 5% CO_2_; cells that did not migrate were removed from the top of the membrane chamber, and those in the membrane were recovered using 150 μL of the cell detachment solution. The eluate (75 μL) was combined with 75 μL of cells that migrated in the chemoattractant solution. The cells were then lysed, and the total number of transmigrated cells was quantified using CyQuant^®^ GR Dye (Synergy™ HT, BioTek, Inc., Winooski, VT, USA), as described by the manufacturer. Cells in RPMI-1640 containing 1% BSA were used as blanks for each chemokine. The remaining cells (150 μL) were used to analyze the CD3^−^ and CD3^+^ MDM subpopulations by flow cytometry. The migration assay was performed in triplicate using MDMs from three healthy donors.

### 4.8. Statistical Analysis

Data are shown as median values and interquartile ranges (IQRs, 10–90). Kruskal–Wallis tests followed by Dunn’s multiple comparisons tests were used. Values of *p* < 0.05 were considered statistically significant (GraphPad Software, Inc., San Diego, CA, USA).

## 5. Conclusions 

**Figure 7:**
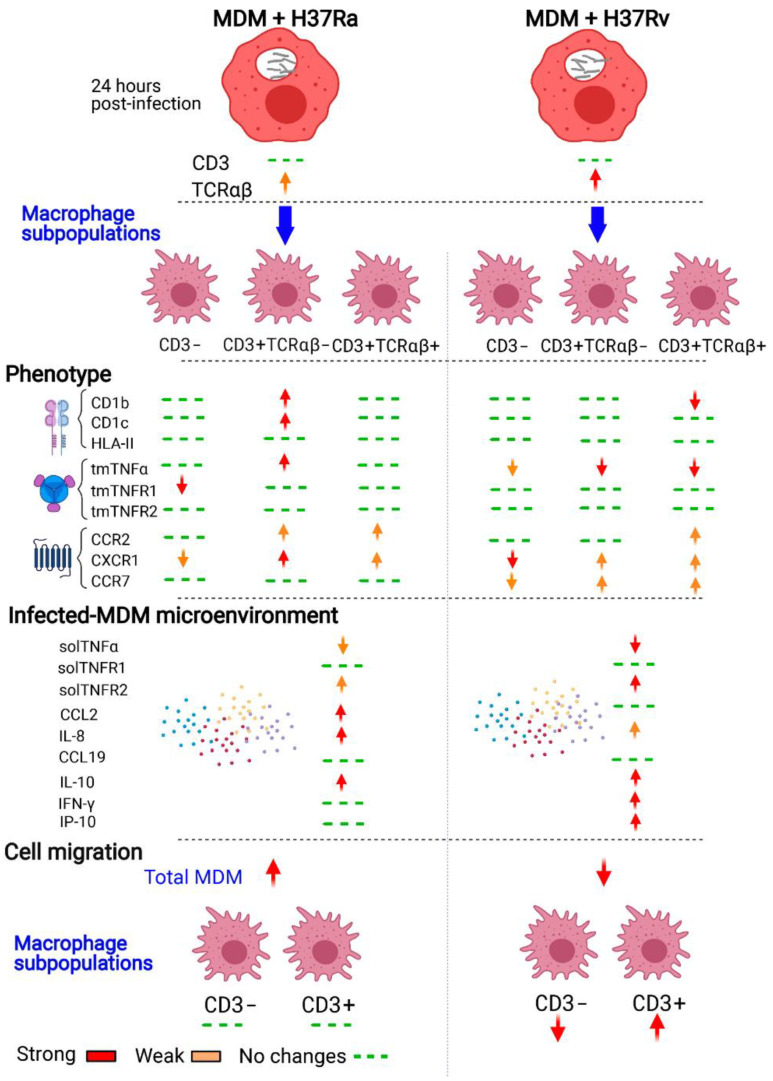
Schematic representation of the immune response of CD3^+^TCRαβ^+^ and CD3^+^TCRαβ^−^ myocyte-derived macrophage (MDM) subpopulations to *Mycobacterium tuberculosis* (Mtb) infection. Human monocytes that are differentiated into macrophages and then infected with the H37Ra or H37Rv Mtb strains display a different phenotype and cytokine microenvironments in response to the infection. CD3^+^TCRαβ^+^ and CD3^+^TCRαβ^−^ MDM subpopulations show differential expression of molecules of the TNF/TNFRs axis, antigen-presenting molecules of MHC class II, and the CD1 family (of protein and lipid, respectively), and chemokine receptors depending on Mtb virulence. The immunological profile generated by H37Rv infection affects the migration ability of MDMs, but interestingly, the migration ability of CD3^−^ MDMs is decreased, whereas that of CD3^+^ MDMs remains intact. The changes are indicated when both MOIs modified the phenotype to simplify the summary of the characterization.

The heterogeneous phenotypes of MDMs generated in response to mycobacterial infection have been summarized in Figure 7. Our data show that the virulent Mtb strain, H37Rv, did not affect the frequency of the cells of the CD3^+^ MDM subpopulation; however, 24 h of infection was enough to increase the expression of TCRαβ in CD3^+^ MDMs. Moreover, infection with the H37Rv virulent strain increased the expression of antigen-presenting molecules on the surface of CD3^+^ MDMs and favored a proinflammatory environment. Its virulence decreased the expression of chemokine receptors on CD3^+^ MDMs, but not the chemokine environment, and CD3^+^ MDMs maintain their migration ability. This study provides evidence that CD3^+^ MDMs might play an essential role in the immune response against Mtb and that the virulence of Mtb might induce the selective recruitment of CD3^+^ macrophages. 

## Data Availability

The authors confirm that the raw data supporting the conclusions of this study are included in the manuscript. The corresponding author will provide more information, upon reasonable request, to any qualified researcher.

## Supplementary Materials

The following are available online at https://www.mdpi.com/article/10.3390/ijms23010329/s1.

## References

[1] World Health Organization (WHO) (2021). Global Tuberculosis Report. https://www.who.int/teams/global-tuberculosis-programme/tb-reports/global-tuberculosis-report-2021.

[2] Cho S.J., Stout-Delgado H.W. (2020). Aging and Lung Disease. Annu. Rev. Physiol..

[3] Sica A., Mantovani A. (2012). Macrophage Plasticity and Polarization: In Vivo Veritas. J. Clin. Investig..

[4] Mantovani S. (2012). Mphage_M1-M2_rev_JCI2012. J. Clin. Investig..

[5] Beham A.W., Puellmann K., Laird R., Fuchs T., Streich R., Breysach C., Raddatz D., Oniga S., Peccerella T., Findeisen P. (2011). A TNF-Regulated Recombinatorial Macrophage Immune Receptor Implicated in Granuloma Formation in Tuberculosis. PLoS Pathog..

[6] Ruiz A., Palacios Y., Garcia I., Chavez-Galan L. (2021). Transmembrane TNF and Its Receptors TNFR1 and TNFR2 in Mycobacterial Infections. Int. J. Mol. Sci..

[7] Rodriguez-Cruz A., Vesin D., Ramon-Luing L., Zuñiga J., Quesniaux V.F.J., Ryffel B., Lascurain R., Garcia I., Chávez-Galán L. (2019). CD3+ Macrophages Deliver Proinflammatory Cytokines by a CD3- and Transmembrane TNF-Dependent Pathway and Are Increased at the BCG-Infection Site. Front. Immunol..

[8] Chavez-Galan L., Vesin D., Blaser G., Uysal H., Benmerzoug S., Rose S., Ryffel B., Quesniaux V.F.J., Garcia I. (2019). Myeloid Cell TNFR1 Signaling Dependent Liver Injury and Inflammation upon BCG Infection. Sci. Rep..

[9] Ocaña-Guzmán R., Téllez-Navarrete N.A., Ramón-Luing L.A., Herrera I., de Ita M., Carrillo-Alduenda J.L., Choreño-Parra J.A., Medina-Quero K., Zúñiga J., Chávez-Galán L. (2021). Leukocytes from Patients with Drug-Sensitive and Multidrug-Resistant Tuberculosis Exhibit Distinctive Profiles of Chemokine Receptor Expression and Migration Capacity. J. Immunol. Res..

[10] Ordway D., Henao-Tamayo M., Harton M., Palanisamy G., Troudt J., Shanley C., Basaraba R.J., Orme I.M. (2007). The Hypervirulent Mycobacterium Tuberculosis Strain HN878 Induces a Potent TH1 Response Followed by Rapid Down-Regulation. J. Immunol..

[11] Park H.-S., Back Y.W., Jang I.-T., Lee K.-I., Son Y.-J., Choi H.-G., Dang T.B., Kim H.-J. (2021). Mycobacterium Tuberculosis Rv2145c Promotes Intracellular Survival by STAT3 and IL-10 Receptor Signaling. Front. Immunol..

[12] Refai A., Gritli S., Barbouche M.R., Essafi M. (2018). Mycobacterium Tuberculosis Virulent Factor ESAT-6 Drives Macrophage Differentiation toward the pro-Inflammatory M1 Phenotype and Subsequently Switches It to the Anti-Inflammatory M2 Phenotype. Front. Cell. Infect. Microbiol..

[13] Feng S., Hong Z., Zhang G., Li J., Tian G.B., Zhou H., Huang X. (2021). Mycobacterium PPE31 Contributes to Host Cell Death. Front. Cell. Infect. Microbiol..

[14] Moody D.B., Guy M.R., Grant E., Cheng T.Y., Brenner M.B., Besra G.S., Porcelli S.A. (2000). CD1b-Mediated T Cell Recognition of a Glycolipid Antigen Generated from Mycobacterial Lipid and Host Carbohydrate during Infection. J. Exp. Med..

[15] de Libero G., Mori L. (2014). The T-Cell Response to Lipid Antigens of Mycobacterium Tuberculosis. Front. Immunol..

[16] Flynn J.L., Chan J. (2001). Immunology of Tuberculosis. Annu. Rev. Immunol..

[17] Domingo-Gonzalez R., Prince O., Cooper A., Khader S.A. (2016). Cytokines and Chemokines in Mycobacterium Tuberculosis Infection. Microbiol. Spectr..

[18] Méndez-Samperio P. (2008). Expression and Regulation of Chemokines in Mycobacterial Infection. J. Infect..

[19] Fuchs T., Puellmann K., Hahn M., Dollt C., Pechlivanidou I., Ovsiy I., Kzhyshkowska J., Gratchev A., Fleig J., Emmert A. (2013). A Second Combinatorial Immune Receptor in Monocytes/Macrophages Is Based on the TCRγδ. Immunobiology.

[20] Fuchs T., Puellmann K., Emmert A., Fleig J., Oniga S., Laird R., Heida N.M., Schäfer K., Neumaier M., Beham A.W. (2015). The Macrophage-TCRαβ Is a Cholesterol-Responsive Combinatorial Immune Receptor and Implicated in Atherosclerosis. Biochem. Biophys. Res. Commun..

[21] McClean C.M., Tobin D.M. (2016). Macrophage Form, Function, and Phenotype in Mycobacterial Infection: Lessons from Tuberculosis and Other Diseases. Pathog. Dis..

[22] Lopez K., Iwany S.K., Suliman S., Reijneveld J.F., Ocampo T.A., Jimenez J., Calderon R., Lecca L., Murray M.B., Moody D.B. (2020). CD1b Tetramers Broadly Detect T Cells That Correlate with Mycobacterial Exposure but Not Tuberculosis Disease State. Front. Immunol..

[23] Layton E.D., Yu K.K.Q., Smith M.T., Scriba T.J., de Rosa S.C., Seshadri C. (2018). Validation of a CD1b Tetramer Assay for Studies of Human Mycobacterial Infection or Vaccination. J. Immunol. Methods.

[24] Liu Y., Wang R., Jiang J., Cao Z., Zhai F., Sun W., Cheng X. (2018). A Subset of CD1c + Dendritic Cells Is Increased in Patients with Tuberculosis and Promotes Th17 cell Polarization. Tuberculosis.

[25] To K.W., Reino J.J.G., Yoo D.H., Tam L.S. (2013). Tumour Necrosis Factor Antagonist and Tuberculosis in Patients with Rheumatoid Arthritis: An Asian Perspective. Respirology.

[26] Keeton R., Allie N., Dambuza I., Abel B., Hsu N.J., Sebesho B., Randall P., Burger P., Fick E., Quesniaux V.F.J. (2014). Soluble TNFRp75 Regulates Host Protective Immunity against Mycobacterium Tuberculosis. J. Clin. Investig..

[27] Cooper A.M., Solache A., Khader S.A. (2007). Interleukin-12 and Tuberculosis: An Old Story Revisited. Curr. Opin. Immunol..

[28] Juffermans N.P., Dekkers P.E.P., Peppelenbosch M.P., Speelman P., van Deventer S.J.H., van der Poll T. (2000). Expression of the Chemokine Receptors CXCR1 and CXCR2 on Granulocytes in Human Endotoxemia and Tuberculosis: Involvement of the P38 Mitogen-Activated Protein Kinase Pathway. J. Infect. Dis..

[29] Cui K., Ardell C.L., Podolnikova N.P., Yakubenko V.P. (2018). Distinct Migratory Properties of M1, M2, and Resident Macrophages Are Regulated by Adβ2and Amβ2integrin-Mediated Adhesion. Front. Immunol..

[30] Chávez-Galán L., Ocaña-Guzmán R., Torre-Bouscoulet L., García-De-Alba C., Sada-Ovalle I. (2015). Exposure of Monocytes to Lipoarabinomannan Promotes Their Differentiation into Functionally and Phenotypically Immature Macrophages. J. Immunol. Res..

[31] Carranza C., Juárez E., Torres M., Ellner J.J., Sada E., Schwander S.K. (2006). Mycobacterium Tuberculosis Growth Control by Lung Macrophages and CD8 Cells from Patient Contacts. Am. J. Respir. Crit. Care Med..

[32] Al-haidari A.A., Syk I., Jirström K., Thorlacius H. (2013). CCR4 Mediates CCL17 (TARC)-Induced Migration of Human Colon Cancer Cells via RhoA/Rho-Kinase Signaling. Int. J. Colorectal Dis..

[33] Campanella G.S.v., Luster A.D. (2009). A Chemokine-Mediated In Vivo T-Cell Recruitment Assay Gabriele. Methods Enzymol..

[34] Carr M.W., Roth S.J., Luther E., Rose S.S., Springer T.A. (1994). Monocyte Chemoattractant Protein 1 Acts as a T-Lymphocyte Chemoattractant. Proc. Natl. Acad. Sci. USA.

