# Supplementary material for: Mycobacterium tuberculosis H37Rv Strain Increases the Frequency of CD3+TCR+ Macrophages and Affects Their Phenotype, but Not Their Migration Ability
Source: Int J Mol Sci. 2021 Dec 28;23(1):329. doi: 10.3390/ijms23010329 (PMC8745617; doi:10.3390/ijms23010329)
Supplement: Supplementary file 1 [file ijms-23-00329-s001.zip › ijms-1456489-supplementary.pdf]

Supplementary Material

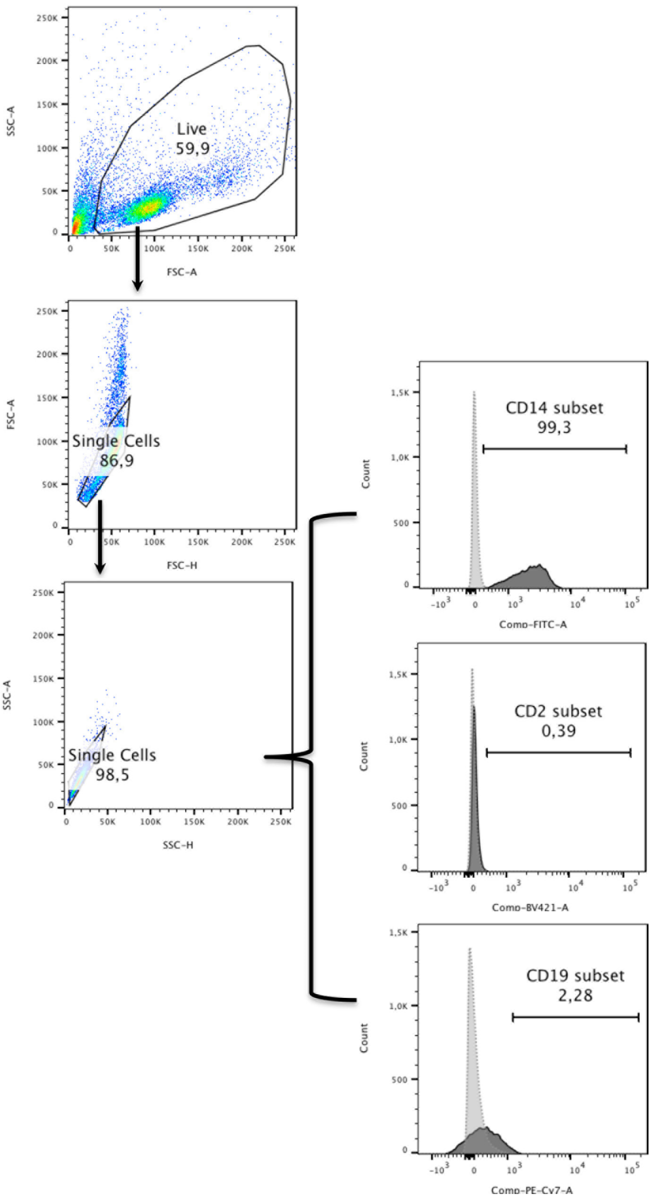

Figure S1. Analysis strategy used to identify the percentage of CD14⁺ purified cells. CD14⁺ purified cells were obtained from PBMCs using a pre-enrichment step by adherence of myeloid cells, followed by a positive immunoselection method with magnetic beads-labelled CD14 antibody. Representative histograms of the CD14⁺ cells purity percentage, and presence of lymphoid cells, evaluated by flow cytometry. Data are representative of at least three independent donors.

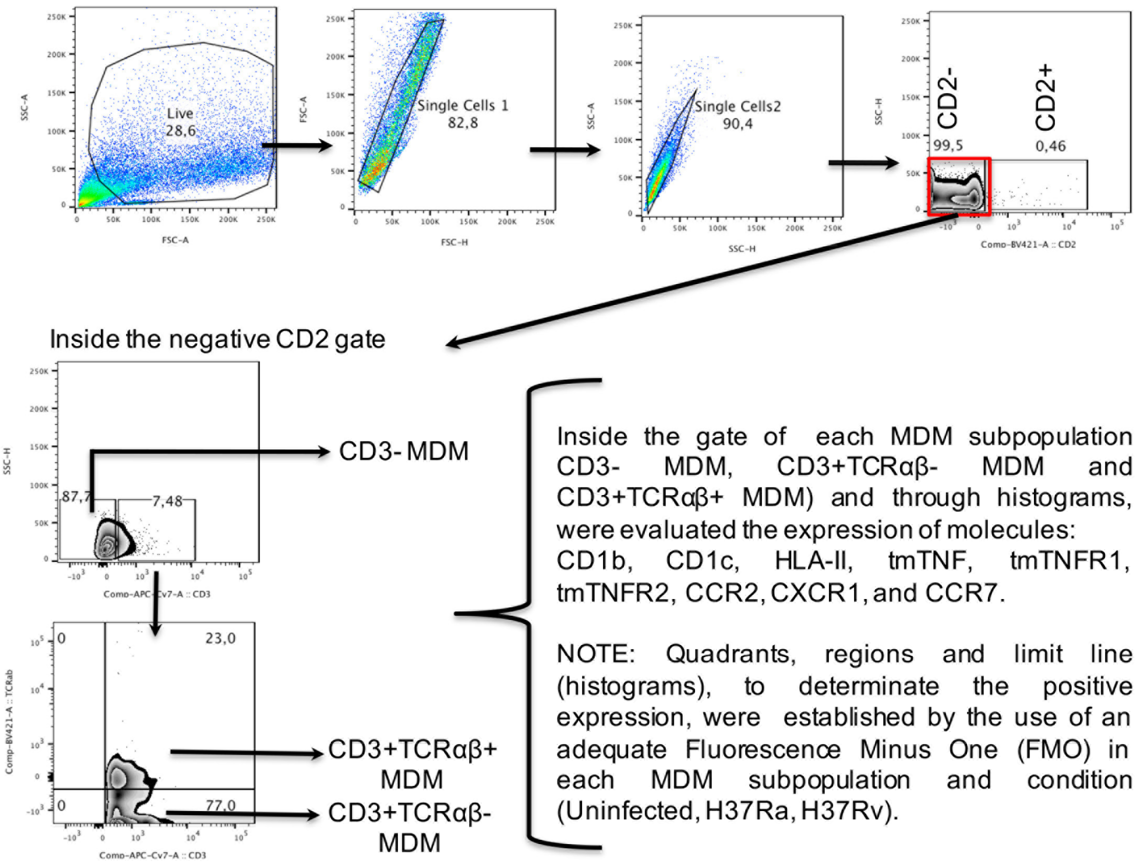

Figure S2. Analysis strategy used to identify the phenotypical characterization of MDM subpopulation recovered after *in vitro* infection. MDM infected with H37Ra (MOI 1 and 10) and H37Rv (MOI 1 and 5) Mtb strains were recovered 24 hours post-culture to evaluate the expression of molecules inside each MDM subpopulation.

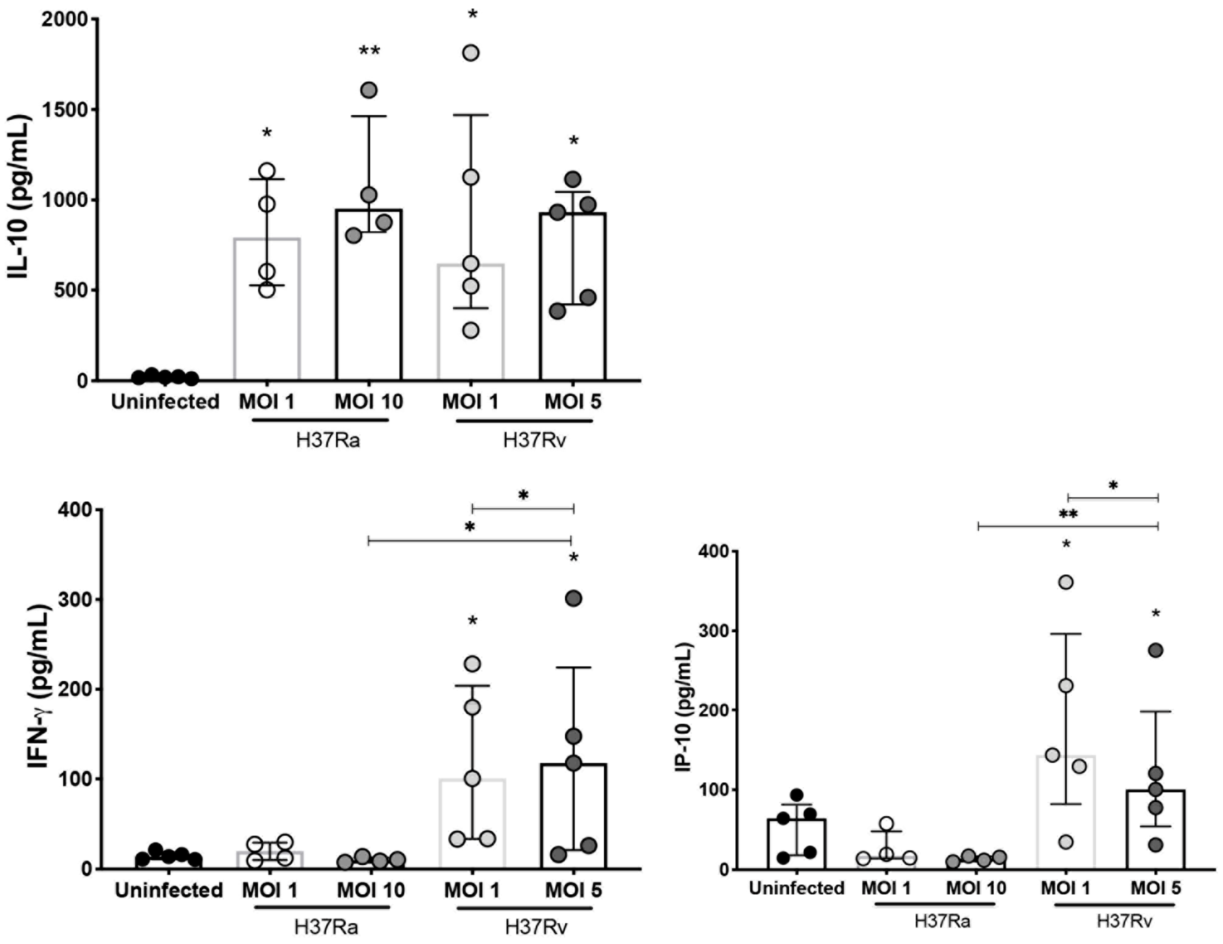

Figure S3. Soluble levels of IL-10, IFN-γ, and IP-10 delivered by MDM infected with H37Ra (MOI 1 and 10) and H37Rv (MOI 1 and 5) Mtb strains. Supernatants were recovered 24 hours post-culture infection to perform ELISA.

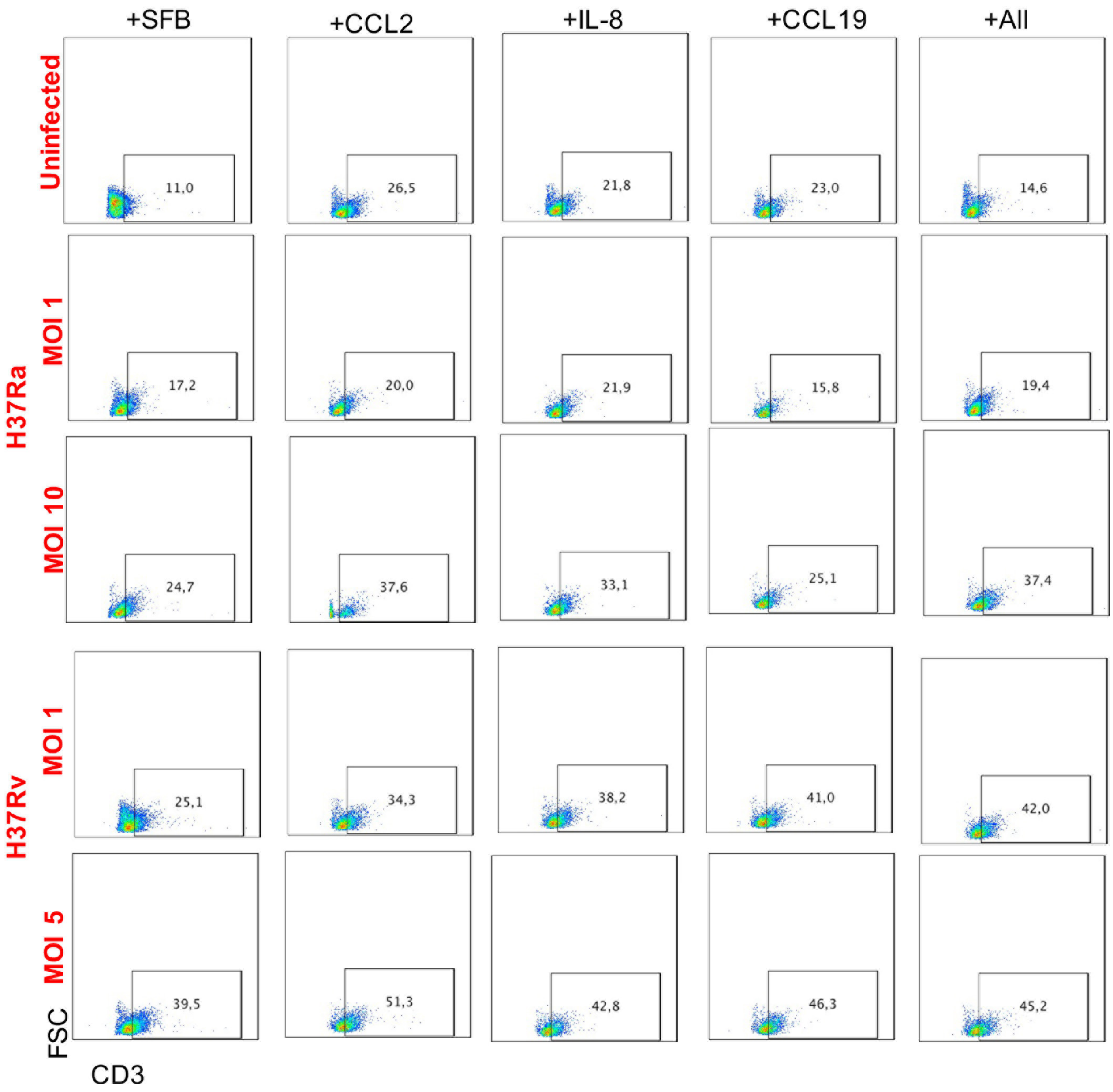

Figure S4. Frequency of $CD3^+$ MDM subpopulation, which migrated in response to CCL2, IL-8, and CCL19, after H37Ra and H37Rv *in vitro* infection. MDM able to migrate chemoattracted by each chemokine or altogether were recovered to analyze $CD3^-$ and $CD3^+$ MDM subpopulations by flow cytometry.

Table S1. List of antibodies used for flow cytometry analysis.

Antibody	Fluorochrome	Clone	Company
CD14	FITC, BV510	HCD14, M5E2	Biolegend
CD2	BV421	TS1/8	Biolegend
CD19	PE Cy7	HIB19	Biolegend
CD3	APC-Cy7	OKT3	Biolegend
TCRαβ	BV421	IP26	Biolegend
HLA-II	APC	L243	Biolegend
CD1b	FITC	SN13	Biolegend
CD1c	PerCP Cy5.5	L161	Biolegend
TNF	APC	MAb11	Biolegend
TNFR1	PE	W15099A	Biolegend
TNFR2	APC	3G7A02	Biolegend
CXCR1	PE Cy7	8F1/CXCR1	Biolegend
CCR2	PE	K036C2	Biolegend
CCR7	APC	G043H7	Biolegend
